# Translating priority effects and niche engineering into rational microbiome therapeutics across the gut-lung axis

**DOI:** 10.3389/fmicb.2026.1871211

**Published:** 2026-06-25

**Authors:** Huimin Chen, Shu Zhang, Yang Bai, Lijie Yu, Ye Gu

**Affiliations:** 1 Department of Pediatric Surgery, The Second Hospital of Jilin University, Changchun, China; 2 Lishu County Traditional Chinese Medicine Hospital, Siping, China

**Keywords:** alveolar macrophage, fecal microbiota transplantation, microbiome, respiratory infections, short-chain fatty acids

## Abstract

The homeostasis of the human microbiome relies on “colonization resistance” governed by complex ecological rules. However, severe perturbations such as broad-spectrum antibiotics can dismantle this defense, shifting the microbial community into a “dysbiotic trap” driven by pathogen niche construction—an alternative stable state that is notoriously difficult to spontaneously reverse. This ecological mechanism explains the frequent failure of empirical therapies like fecal microbiota transplantation (FMT) and blind probiotic supplementation. Crucially, local ecological collapse triggers systemic cascades via the “gut-lung axis.” The depletion of core gut metabolites, such as short-chain fatty acids, impairs the metabolic reprogramming and antimicrobial capacity of distal alveolar macrophages. This cascade drastically increases host susceptibility to respiratory infections. To break this clinical deadlock, microbiome medicine must transition from “empirical transplantation” to “rational microbiome engineering.” This review systematically outlines the core pillars of this translational framework: achieving “precision niche clearing” via targeted bacteriophages; capturing optimal intervention windows to harness “priority effects”; and ultimately engrafting “synthetic microbial consortia” (SMCs) rationally designed upon metabolic cross-feeding principles. This strategy offers a promising avenue to durably shatter the dysbiotic deadlock and restore host immune homeostasis across the gut and systemic levels.

## 1 Introduction

Over the past decade, the rapid development of multi-omics technologies has immensely expanded the biomedical community’s cognitive boundaries regarding the human microbiome. These technologies include metagenomics, transcriptomics, and metabolomics (Schokker et al., 2025). The focus of scientific inquiry has decisively transcended the early census stage of cataloging “who is there”. It has now entered a far more complex and profound frontier: deciphering the internal operational mechanics of microbial communities, namely, “the rules of assembly and dynamics” (Schokker et al., 2025). The human gastrointestinal tract is an exceedingly crowded bioreactor characterized by fierce resource competition, harboring trillions of microorganisms. Having undergone prolonged co-evolution with the host, these symbiotic consortia facilitate deep nutrient metabolism. They also play an indispensable, foundational role in immune system maturation and defense against pathogen invasion (Chang, 2020).

However, the ecological characteristics of the microbiome present a highly challenging clinical paradox. In a state of health, the microbiome exhibits formidable ecological resilience and resistance to perturbations. By establishing intricate metabolic cross-feeding networks and monopolizing all available ecological niches, it constructs an robust ecological barrier that effectively excludes potential pathogens (Caballero-Flores et al., 2023). Yet, once this ecological equilibrium is subjected to severe disruptions, such as repeated broad-spectrum antibiotic exposure or severe infections, the original homeostatic network suffers a profound disruption (Ming and Ray, 2019; Suvvari et al., 2025). Problematic still, the dismantled microbiome rarely reverts to its baseline health spontaneously. Instead, it frequently reassembles and stabilizes into a novel, pathobiont-dominated community structure. In theoretical ecology, this phenomenon is termed “alternative stable states” (Garza et al., 2025). Once the system transitions into this pathological alternative stable state—the “dysbiotic trap”—it exhibits remarkable stability, persisting against medical interventions aimed at restoring health (Winter et al., 2013).

This ecological impasse profoundly explains the limitations of current microbiome-targeted therapies. Conventional Fecal Microbiota Transplantation (FMT) has achieved remarkable success in treating recurrent *Clostridioides difficile* infection (rCDI). However, its efficacy becomes highly unpredictable and transient when addressing more complex chronic dysbiotic traps, such as inflammatory bowel disease (IBD), obesity, or metabolic syndrome (Cappio Barazzone et al., 2024). Similarly, empirical administration of generic probiotics often results in engraftment failure. The exogenous strains are swiftly excluded by the incumbent community because they fail to fundamentally dismantle the pathological niches already firmly occupied by pathobionts (Cappio Barazzone et al., 2024).

**FIGURE 1:**
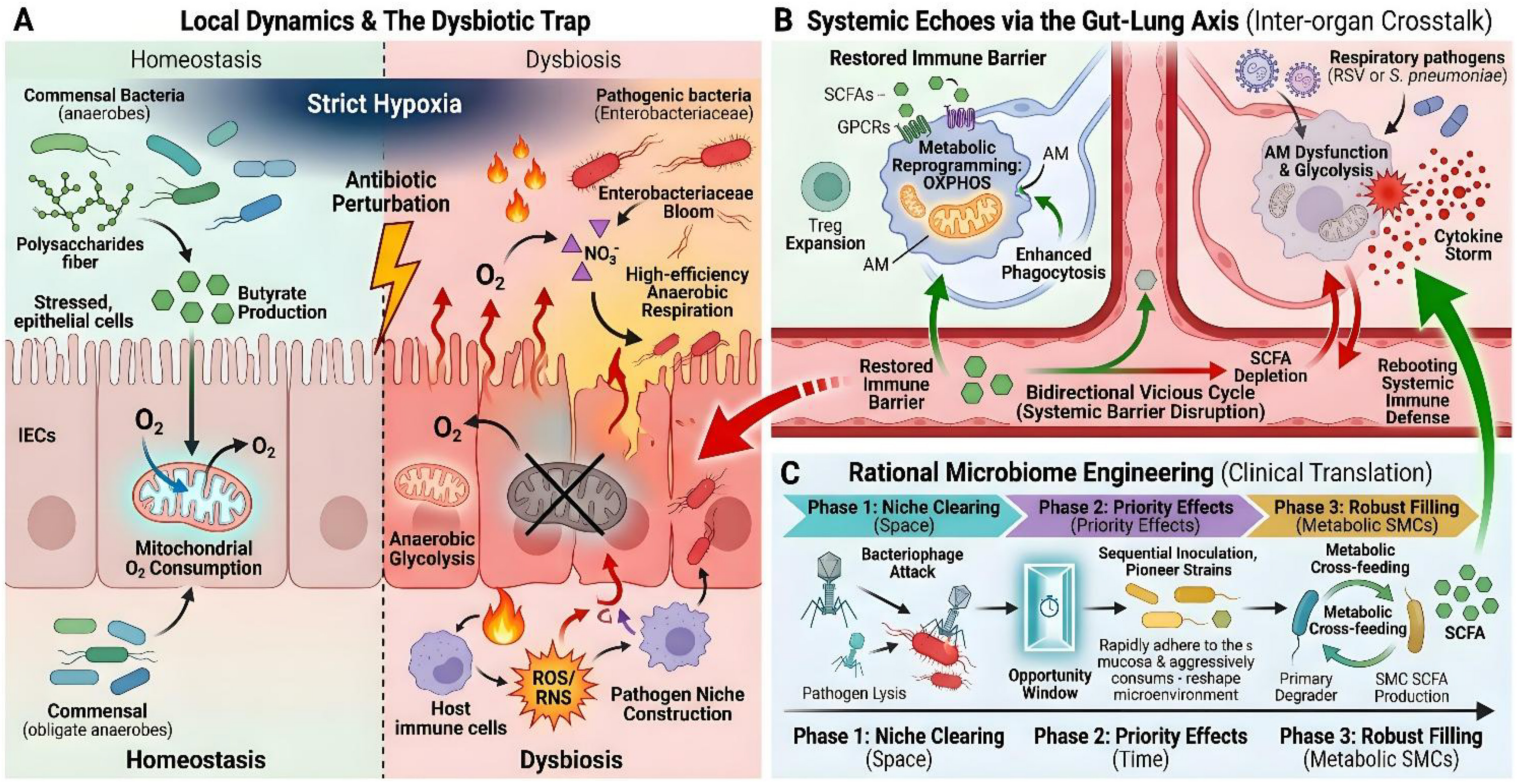
Deconstructing the dysbiotic trap and rational microbiome engineering across the gut-lung axis. **(A)** Local ecological dynamics and the “dysbiotic trap”. Under homeostatic conditions (left), commensal obligate anaerobes degrade polysaccharides to produce butyrate, which fuels colonocyte mitochondrial β-oxidation. This profound oxygen consumption maintains strict luminal hypoxia and robust colonization resistance. Following severe perturbation (e.g., broad-spectrum antibiotic exposure, indicated by the lightning bolt, right), commensal depletion forces colonocytes to shift toward anaerobic glycolysis, causing luminal molecular oxygen leakage. Concurrently, host mucosal inflammation generates ROS/RNS, yielding terminal electron acceptors such as nitrate (NO^3–^). Facultative anaerobic pathobionts (e.g., *Enterobacteriaceae*, red) exploit these substrates via high-energy anaerobic respiration, actively driving pathogen niche construction and locking the local ecosystem into an intractable alternative stable state—the “dysbiotic trap.” **(B)** Systemic immune echoes via the gut-lung axis. In health (green arrows), gut-derived SCFAs (e.g., acetate and propionate) enter the systemic circulation and bind to specific receptors on AMs. This metabolic signaling drives a profound reprogramming toward mitochondrial OXPHOS, optimizing AM phagocytic capacity and promoting local FOXP3^+^ Treg expansion. Conversely, during gut dysbiosis (red arrows), profound SCFA depletion forces AMs to default to inefficient glycolysis. Upon respiratory pathogen invasion (e.g., RSV, *S. pneumoniae*), this metabolic paralysis precipitates a catastrophic cytokine storm. The ensuing systemic inflammatory signals retrogradely compromise the intestinal barrier, fueling a bidirectional vicious cycle of multi-organ vulnerability. **(C)** Spatiotemporal framework for rational microbiome engineering. A spatiotemporally coordinated, three-step interventional timeline designed to permanently dismantle the dysbiotic trap. Step 1 (Niche Clearing): Strictly lytic bacteriophages act as precision biological scalpels to specifically lyse targeted pathobionts, liberating physical and nutritional niches without collateral damage. Step 2 (Priority Effects): Within this engineered window of opportunity, pioneer strains are sequentially inoculated to rapidly consume residual oxygen and favorably reshape the local microenvironment. Step 3 (Metabolic SMC Engraftment): SMCs are engrafted, rationally structured upon metabolic cross-feeding networks between primary degraders and secondary fermenters. The robust restoration of SCFA production [thick arrow pointing to panel **(B)**] directly signals back to the pulmonary compartment, reawakening systemic immune defenses and durably restoring cross-organ homeostasis. AM, alveolar macrophage; OXPHOS, oxidative phosphorylation; ROS/RNS, reactive oxygen/nitrogen species; RSV, respiratory syncytial virus; SCFA, short-chain fatty acid; SMC, synthetic microbial consortium; Treg, regulatory T cell.

Furthermore, microbiome assembly and dysbiosis are by no means confined to their localized anatomical compartments. The assembly trajectories of the local gut microbiome release massive quantities of metabolites and immunomodulatory factors into the systemic circulation, serving as systemic signals for “environmental filtering” in distal organs (Marrella et al., 2024). Particularly within the “gut-lung axis” (GLA), the depletion of specific metabolites caused by gut ecological collapse can directly impair the defensive functions of pulmonary immune cells. This impairment triggers bidirectional disease susceptibility (Verma et al., 2024). Therefore, the central thesis of this review is to propose a translational breakthrough: modern microbiome medicine must transcend the limitations of the empirical “black box” intervention model. Developing truly transformative “rational microbiome engineering” interventions requires deeply comprehending and mastering higher-order ecological rules. These rules include niche construction, the dynamic capture of priority effects, and cross-organ metabolic communication. Mastering these principles is essential to dismantle the dysbiotic trap and achieve durable host homeostasis (Figure 1).

## 2 The ecological anatomy of colonization resistance and the “dysbiotic trap”

Understanding why microbiome dysbiosis is notoriously difficult to reverse requires a precise deconstruction of colonization resistance. This defense is an ecological fortress co-constructed by the highly coordinated synergy between the microbial community and the host immune system (Chang, 2020).

### 2.1 Niche preemption & host filtering

During physiological homeostasis, the commensal microbiota (predominantly composed of *Bacteroidota* and *Bacillota*) establishes deterministic colonization resistance through a strategy of extreme “niche preemption” (Caballero-Flores et al., 2023). This strategy is primarily manifested in the highly efficient and comprehensive monopolization of core nutrients. Resident keystone taxa are equipped with massive, highly specialized Polysaccharide Utilization Loci (PULs). These loci enable them not only to rapidly degrade complex dietary fibers but also to directly utilize host-secreted mucin and sialic acid as carbon substrates for colonization (Safarchi et al., 2025). By driving these basal nutritional resources below the threshold required for pathogen growth, the commensal community physically and metabolically deprives potential invaders.

In addition, the commensal community directly eliminates competitors by secreting an array of antimicrobial compounds (e.g., bacteriocins). They also engage in contact-dependent interspecies antagonism via Type VI Secretion Systems (T6SS; Jones et al., 2025). Crucially, primary microbial metabolites—especially short-chain fatty acids (SCFAs) like butyrate, propionate, and acetate—significantly lower the local luminal pH, creating an acidic microenvironment intolerable to most enteric pathogens (Chang, 2020).

In this process, the host forms a strict alliance with commensals through “host filtering.” Microbial metabolic signals stimulate goblet cells in the intestinal epithelium to secrete massive amounts of mucus, fortifying the physical barrier. Simultaneously, they stimulate lamina propria B cells to secrete secretory IgA (sIgA) and induce epithelial cells to express potent antimicrobial peptides (AMPs), such as C-type lectins (e.g., Reg3γ) and defensins (Safarchi et al., 2025). These immune effectors precisely sequester invading pathogens outside the mucus layer, while commensals have acquired profound tolerance to these host defenses over long evolutionary periods.

### 2.2 Perturbation and the emergence of alternative stable states

While powerful, colonization resistance is not invincible. When the host experiences severe systemic perturbations, such as broad-spectrum antibiotic treatment, the dense ecological network is instantaneously torn apart (Ng et al., 2019). While killing target pathogens, antibiotics often indiscriminately eradicate highly sensitive anaerobic commensals, causing a precipitous drop in species diversity (Xue et al., 2023). This sudden population crash creates a massive “ecological vacuum,” instantly releasing free carbohydrates, amino acids, and host glycans that were previously tightly controlled by commensals (Garza et al., 2025).

Based on classical ecological theory, once external interference is removed, the community should undergo secondary succession and gradually return to its original steady state. However, microbial communities frequently exhibit “multistability” because they are highly complex non-linear dynamical systems (Garza et al., 2025). Extensive empirical research includes rigorous validations in rat intestinal models and synthetic human gut microbial *in vitro* models. These studies have conclusively demonstrated that the gut microbiome can assemble and stabilize into fundamentally different community configurations under identical external environmental conditions (Van de Guchte et al., 2020).

Severe perturbations like antibiotics can push the system past an imperceptible ecological “tipping point.” Consequently, the entire community undergoes a phase transition along its energy landscape, shifting into an “alternative stable state” dominated by pathobionts (Fujita et al., 2025). In this profoundly disrupted state, even after the pharmacological pressure of antibiotics has dissipated, the newly formed pathological network sustains itself through aberrant metabolic interactions, exhibiting formidable pathological resilience (Garza et al., 2025). This is the ecological root of why patients subjected to heavy medical interventions often experience intestinal dysbiosis that persists for months or years without spontaneous recovery.

### 2.3 Host metabolic reprogramming and pathogen niche construction

The deeper issue lies in the fact that this alternative stable state is an active environment established and maintained by opportunistic invading pathogens. This process is ecologically termed “niche construction” (Caballero-Flores et al., 2023). In a typical dysbiotic trap, facultative anaerobic *Enterobacteriaceae* (such as *Escherichia coli* and *Salmonella Typhimurium*) display formidable metabolic flexibility, becoming the dominant forces in this pathological steady state (Caballero-Flores et al., 2023).

In a healthy gut, epithelial cells consume massive amounts of microbially-produced butyrate via mitochondrial β-oxidation. This process severely depletes oxygen near the epithelium, thereby maintaining a strictly hypoxic physiological microenvironment (Moreira de Gouveia et al., 2024). However, when antibiotics cause a mass extinction of butyrate producers, colonocytes are forced to undergo metabolic reprogramming, shifting their energy source from mitochondrial β-oxidation to anaerobic glycolysis (Moreira de Gouveia et al., 2024). The subsequent drastic decline in epithelial oxygen consumption allows molecular oxygen to leak into the gut lumen (Moreira de Gouveia et al., 2024).

The emergence of oxygen triggers host inflammation. Immune macrophages and neutrophils recruited to the mucosa release high concentrations of reactive oxygen species (ROS) and reactive nitrogen species (RNS) to combat pathogens (Liou et al., 2022). These free radicals react with luminal compounds to generate oxidative byproducts, most notably nitrate (Spees et al., 2013).

This marks the critical moment when pathogens construct their niche. Unlike obligate anaerobes restricted to inefficient fermentation, facultative anaerobic *Enterobacteriaceae* have evolved highly sophisticated sensory and metabolic systems. They rapidly upregulate the expression of various terminal reductases (such as nitrate reductases *narGHJI*, *napFDAGHBC*) (Moreira de Gouveia et al., 2024). By utilizing host inflammation-derived nitrate as a highly efficient electron acceptor, pathogens initiate a high-energy-yielding “anaerobic respiration” mode (Winter et al., 2013). Not only do they actively seek out these electron-acceptor-rich inflammatory niches via chemotaxis (Rivera-Chávez et al., 2013), but they also continuously “manufacture” their exclusive nutrients by provoking sustained mucosal inflammation (Caballero-Flores et al., 2023).

In this detrimental feedback loop, pathogen-induced inflammation maintains the nitrate supply, while high-yield nitrate respiration allows pathogens to quantitatively outcompete the slowly recovering commensals, establishing their own “pathological colonization resistance.” This ecological niche-constructing behavior—exploiting the host’s inflammatory response to acquire exclusive nutrients—is the absolute core mechanism rendering the “dysbiotic trap” physiologically impenetrable to spontaneous recovery (Spees et al., 2013).

## 3 Systemic echoes: assembly rules dictating the gut-lung axis

The mammalian mucosal immune system is a highly interconnected global network. The assembly and collapse trajectories of microbes within the vast habitat of the gut are by no means merely local ecological events. Instead, they inevitably generate widespread “systemic echoes”. These act as potent environmental filtering signals, crossing anatomical boundaries to profoundly dictate and reshape the physiological functions and disease susceptibility of distal organs (Marrella et al., 2024). Among the various inter-organ axes, the gut-lung axis (GLA) is not only the most extensively studied but also perfectly exemplifies how local microbial ecological networks dictate global host resilience (Verma et al., 2024).

### 3.1 Cross-anatomical ecological and metabolic communication

The gut microbiome effectively operates as a highly active endocrine organ within the host (Wang et al., 2025). The key mediators sustaining gut-lung communication are the SCFAs produced by keystone commensals. High concentrations of acetate and propionate can breach the intestinal barrier, bypass complete hepatic first-pass elimination, and enter the systemic circulation. They successfully reach physiological micro-molar concentrations within the highly vascularized capillary beds of the lungs, acting as direct biochemical messengers (Maruyama et al., 2026).

### 3.2 Metabolic reprogramming of alveolar macrophages

Once these gut-derived SCFAs arrive in the pulmonary microenvironment, they are specifically recognized by G-protein-coupled receptors (primarily FFAR2 and FFAR3) densely expressed on Alveolar Macrophages (AMs; Liu et al., 2021). Upon binding, SCFAs trigger a profound metabolic reprogramming. While unchallenged macrophages faced with pathogens typically shift toward inefficient aerobic glycolysis, continuous SCFA nourishment reverses this shift. It pushes their metabolic preference back toward highly efficient mitochondrial oxidative phosphorylation (OXPHOS; Maruyama et al., 2026). This metabolic flexibility drastically enhances the phagocytic and intracellular bactericidal capacity of AMs. Furthermore, SCFAs act as potent Histone Deacetylase Inhibitors (HDACi), promoting the local expansion of FOXP3+ Regulatory T cells (Tregs) to proactively shield alveolar structures from irreversible inflammatory damage (Liu et al., 2025; Sun et al., 2025).

### 3.3 Bidirectional disease susceptibility and the vicious cycle

#### 3.3.1 Gut dysbiosis compromises pulmonary immunity

Given this profound metabolic dependence, the governing role of gut assembly rules over respiratory health is glaringly evident. Extensive clinical data confirms that gut dysbiosis leads to a sudden drop in systemic SCFA levels, instantly leaving the host’s lungs in a state of immune vulnerability (Marrella et al., 2024). Deprived of this crucial metabolic nourishment, AM functions are paralyzed. Consequently, when the host encounters respiratory pathogens (e.g., *Streptococcus pneumoniae* or RSV), pulmonary clearance rates decrease significantly, often accompanied by severe cytokine storms and severe organ dysfunction (Wang et al., 2025).

#### 3.3.2 Respiratory infection aggravates gut dysbiosis

Crucially, this ecological conduit is bidirectional. A severe acute respiratory infection intrinsically acts as a systemic disaster. The massive influx of inflammatory cytokines (such as IL-6, TNF-α, IL-1β, and IFN-γ) surges through the systemic circulation, directly compromising the physical integrity of the gut mucosa (Sencio et al., 2021). Concurrently, pulmonary-induced systemic hypoxia alters the mucosal oxygen gradient in the gut. As detailed in section “2.3 Host metabolic reprogramming and pathogen niche construction,” this influx of oxygen and inflammatory byproducts provides the exact pathological niche required for hypoxia-tolerant facultative anaerobes to thrive. Consequently, strict obligate anaerobes are suppressed, specifically favoring the rapid, secondary expansion of *Enterobacteriaceae* (Dumas et al., 2018; Liu et al., 2025). Regarding the durability of these shifts, while mild dysbiosis may be reversible upon the swift resolution of the respiratory infection, severe or prolonged perturbations can lead to permanent structural ecological alterations. Ultimately, the respiratory infection compromises the gut’s defenses, while the resulting gut collapse depletes the immune resources required by the lungs, locking the host into a severe, self-amplifying cycle of multi-organ inflammatory failure.

## 4 Translating ecology to therapeutics: rational microbiome engineering

Faced with the formidable pathological barriers co-constructed by the “dysbiotic trap” and “gut-lung axis feedback,” attempting to resolve the issue by merely supplementing uncalculated generic probiotics or performing untargeted full-spectrum FMT is wholly inadequate for addressing such profound ecological disruptions. Clinical microbiome science urgently needs to step out of the blind spot of empirical medicine and enter the era of “Rational Microbiome Engineering,” guided by systems dynamics and objective ecological laws (Schokker et al., 2025). This is a systemic reconstructive campaign conducted on a microscopic scale. Its core lies in flexibly commanding the rules of time (harnessing priority effects) and spatial topology (precision niche engineering) to systematically dismantle the pathogen’s niche (Table 1) and permanently engraft a health-sustaining ecological network.

**TABLE 1:** Summary of ecological principles and corresponding rational microbiome therapeutic strategies.

Ecological principle	Mechanism in the microbiome	Translational therapeutic strategy
Niche preemption	Monopolization of resources and spatial colonization sites by early colonizers.	Pioneer strains: Sequential inoculation of engineered strains with rapid mucosal adhesion.
Priority effects	The historical timing and order of species arrival dictate the assembly trajectory.	Timing interventions: Capturing a clinical “window of opportunity” for precisely timed inoculations.
Niche construction	Pathobionts hijack host inflammation to create exclusive metabolic niches (e.g., nitrate).	Phage-mediated niche clearing: Targeted lysis of specific pathobionts without collateral damage.
Cross-feeding	Syntrophic interactions and metabolic interdependence between microbial species.	Synthetic microbial consortia (SMCs): Co-administering primary degraders and secondary fermenters.

### 4.1 Deconstructing the ecological logic and limitations of FMT

For a long time, FMT has been viewed as a potent therapeutic tool of microbiome therapeutics, showing astonishing restorative capabilities, particularly against recurrent infections caused by *C. difficile* (rCDI). However, deconstructing the ecological logic of FMT’s success precisely exposes its fatal shortcomings when treating broader chronic diseases.

The success of FMT in rCDI essentially exploits the “ecological vacuum” created by the pathogen itself. Prolonged antibiotic therapy virtually eradicates intestinal competitors, bringing secondary bile acid metabolism to a complete halt, which sets the perfect stage for *C. difficile* spore germination and vegetative growth. At this point, the introduction of a massive, intact community from a healthy donor (or purified healthy *Bacillota* spore fractions, such as the FDA-approved SER-109) can rapidly accomplish “niche preemption” in the new environment (Khanna et al., 2022). The newly engrafted community instantaneously reboots the secondary bile acid conversion network and provides seamless coverage over space and carbon sources, thereby metabolically “starving” and depriving *C. difficile* of its foothold from the source (Khanna et al., 2022).

However, when attempting to apply this identical FMT strategy to metabolic and immune disorders such as IBD, obesity, or autism, efficacy decreases substantially (Cappio Barazzone et al., 2024). This is because the guts of these patients are not in an “ecological vacuum”; rather, they are deeply entrenched in a “dysbiotic trap” dominated by highly adaptable pathobionts. The residual pathological flora not only maintains dominance through continuous niche construction (e.g., nitrate respiration) but also utilizes robust priority effects and resource blockades to competitively exclude any incoming “donor community” (Xue et al., 2023). Under this extreme “niche mismatch” between donor and recipient, combined with the fierce resistance of the residual pathological network, blind FMT engraftment is highly prone to failure (Cappio Barazzone et al., 2024). Ultimately, without prior niche clearing to eliminate incumbent pathobionts and disrupt their pathological resilience, the donor community cannot successfully colonize. This fundamental ecological barrier explains the limited and transient efficacy of empirical FMT in chronic dysbiotic conditions.

### 4.2 The dimension of time: harnessing priority effects

In the succession of microbial ecosystems, the natural law of “first come, first served” is vividly demonstrated—a concept known in ecology as historical contingency or “priority effects” (Debray et al., 2022). Priority effects explicitly dictate that the order and timing of species arrival into an ecosystem will directly and irreversibly determine the subsequent assembly trajectory and final configuration of the entire community. This effect can be inhibitory (the pioneer strain consumes critical nutrients and occupies binding sites, repelling latecomers) or facilitative (the pioneer strain paves the way for specific later-arriving species by secreting intermediate metabolites or altering the physical environment) (Jones et al., 2025).

Extensive empirical studies—from T6SS-mediated priority colonization by *Snodgrassella alvi* in honeybee gut models to bacterial-yeast competition in floral nectar (*Mimulus*)—have repeatedly confirmed the absolute dominance of priority effects (Jones et al., 2025). In human medicine, to conquer the dysbiotic trap, priority effects must be translated into a precise clinical therapeutic weapon (Debray et al., 2022).

This requires interventions with extreme temporal precision (Timing). The primary task of rational engineering is to find or artificially create an unhindered “window of opportunity” (Law et al., 2024). Such an optimal vacuum period naturally occurs during the infant stage of early life, or can be induced in adult patients following extreme intestinal debridement and antibiotic washout (Pantazi et al., 2023). In clinical practice, determining exactly when a patient has entered this optimal window remains challenging, and standardized diagnostic guidelines are currently lacking. However, to capture this critical intervention period, clinicians could potentially monitor specific dynamic biomarkers. These may include a sharp reduction in fecal SCFA concentrations, a dynamic drop in fecal calprotectin indicating the temporary resolution of acute mucosal inflammation, or specific microbiota shifts (e.g., tracking the obligate-to-facultative anaerobe ratio) measured via rapid 16S rRNA sequencing.

Clinicians no longer blindly administer mixed colonies. Instead, they sequentially introduce rationally engineered and selected “pioneer strains” (Sleator and Hill, 2009). These pioneers must possess exceptional initial growth rates and ultra-strong adhesion to bare mucosa to ensure absolute niche preemption. More importantly, they carry a specific metabolic mission: to induce a potent “facilitative priority effect” by reshaping physical and chemical gradients at the mucosal base (e.g., depleting residual oxygen, lowering pH). Once the pioneer strains establish a foothold, they act as guides for the subsequent healthy microbial community. They safely direct the completion of primary or secondary succession, permanently closing the succession channel before pathogens can resurge (Sprockett et al., 2018).

### 4.3 The dimension of space: precision niche engineering

However, merely seizing the right timing is insufficient. Ensuring the longevity of therapeutic effects requires a fundamental “renovation” of the spatial and metabolic topology within the microbiome. Precision niche engineering consists of two inseparable steps: precision clearing and robust filling.

#### 4.3.1 Niche clearing: biological ultra-narrow-spectrum debridement

In a gut trapped in dysbiosis, key pathobionts (e.g., pro-inflammatory *E. coli* or *Klebsiella*) are deeply embedded within host mucosal folds. Using traditional broad-spectrum antibiotics for debridement is not only ineffective but deepens the dysbiotic basin by inflicting massive collateral damage on commensals (Ng et al., 2019). Therefore, clearing operations demand highly targeted “biological scalpels.” Currently, the most promising weapon is phage therapy (Marrella et al., 2024).

Strictly lytic bacteriophages, viruses capable of specifically infecting and lysing bacteria, exhibit remarkably stringent host specificity. Phage cocktails—constructed via genetic engineering (e.g., CRISPR-Cas modifications) or high-throughput screening—act as highly specific antimicrobial agents within the intestinal environment. They precisely target and lyse specific target pathogens with surgical precision, leaving the thousands of vulnerable commensal colonies entirely unharmed (Singh et al., 2025). This surgical strike not only effectively reduces the pathogen biomass but, more importantly, effectively opens an exclusive, nutrient-rich niche previously monopolized by pathogens within the dense pathological network, clearing the spatial obstacles for subsequent ecological reconstruction (Cappio Barazzone et al., 2024). However, it must be objectively acknowledged that while phage therapy represents a highly promising intervention in theoretical and preclinical models, there is currently a significant lack of large-scale human clinical trials demonstrating its routine efficacy and safety for complex human intestinal diseases.

#### 4.3.2 Metabolic cooperation: synthetic microbial consortia (SMCs) and syntrophy

Once a niche is cleared, if it is not robustly filled immediately, stubborn pathobionts or new opportunistic invaders will rapidly mount a comeback. Therefore, the final and most critical step of the engineering process is the implantation of Synthetic Microbial Consortia (SMCs; Schokker et al., 2025). Unlike therapies relying on natural fecal banks, SMCs are rationally designed microbial consortia rigorously designed through “bottom-up” computational logic based on multi-omics data and metabolic flux networks (McCarty and Ledesma-Amaro, 2019).

The soul of constructing highly efficient SMCs lies in perfectly architecting a stable “metabolic cross-feeding” and syntrophic interaction network (Deter and Lu, 2022). In this design, different strains are assigned strict divisions of labor: primary degraders, armed with powerful complex polysaccharide-degrading capabilities, are co-administered with specific matching prebiotics, ensuring they secure an absolute competitive advantage during the initial colonization phase (Pérez Escriva et al., 2022). The intermediate oligosaccharides or metabolic byproducts (such as succinate) released by primary degraders after cleaving macromolecules serve as the exclusive carbon sources for the second-tier secondary fermenters.

By artificially tethering multiple bacterial strains into an interdependent metabolic assembly line, the entire SMC merges into a highly synergistic “super-organism” (Deter and Lu, 2022). This extreme efficiency of resource utilization not only fills the physical niche but creates an absolute local resource blockade against residual pathogens, metabolically depriving them at the source. Most ingeniously, the ultimate metabolic output of this rigorously designed SMC is deliberately locked into the high-yield production of core SCFAs like butyrate and propionate (Pérez Escriva et al., 2022). These potent metabolic signals not only instantaneously quell local intestinal inflammation but rapidly travel via the systemic circulation directly to the lungs, reawakening the OXPHOS metabolic tone of alveolar macrophages. Thus, from local niche battles in the gut to systemic immune remodeling across the gut-lung axis, rational microbiome engineering thoroughly disrupts the detrimental cycle of the dysbiotic trap (Wang et al., 2025).

### 4.4 Limitations and translational challenges

Although rational microbiome engineering offers a transformative framework, several critical translational challenges must be addressed before widespread clinical application. First, the profound inter-individual variability of the human microbiome and host genetics means that universal “off-the-shelf” synthetic consortia may exhibit inconsistent efficacy across different patients. Second, the clinical application of bacteriophages faces significant pharmacological hurdles, including difficulties in targeted delivery to the lower gastrointestinal tract, maintaining viral stability in acidic environments, and the rapid emergence of phage-resistant bacterial mutants. Third, despite rigorous *in silico* design, the *in vivo* engraftment of Synthetic Microbial Consortia (SMCs) remains highly unpredictable due to complex host-diet-microbiome interactions. Finally, there is currently a distinct lack of large-scale, randomized clinical trials directly demonstrating the efficacy of priority-effect-based interventions and sequential inoculations in human cohorts. Overcoming these hurdles will require advances in personalized diagnostics, improved targeted delivery systems, and rigorous clinical validation.

## 5 Conclusion and future perspectives

Microbiome medicine stands at a historic turning point. To truly translate prolific basic research discoveries into long-lasting, stable clinical therapies, we must decisively transcend the “black-box” paradigm of blind probiotic supplementation and empirical FMT, fully embracing “systems engineering” guided by rigorous, objective ecological laws. The human microbiome is not a random pile of microbes, but a non-equilibrium thermodynamic system maintained by strict assembly rules, potent priority effects, and intricate spatial niche networks. Recognizing that under severe perturbation, the microbiome can transition into a resilient “dysbiotic trap” via pathogen-driven niche construction, is the theoretical prerequisite for developing next-generation rational therapies. Moreover, elevating our perspective to the systemic level of the “gut-lung axis” brings a profound realization. Repairing gut ecological collapse is not merely about alleviating gastrointestinal inflammation. It fundamentally involves rebooting the antibacterial defense network of alveolar macrophages via distal metabolic communication, thereby interrupting the self-amplifying cycle of multi-organ inflammatory failure.

Looking toward the future, the ultimate milestone for achieving extreme personalization and precision in microbiome engineering lies in overcoming the predictive challenges of spatio-temporal evolutionary dynamics. Achieving this goal relies heavily on the deep mining and integration of continuous, densely sampled longitudinal multi-omics data (Zhang et al., 2025a). Historically, to mathematically model these ecological dynamics, researchers have primarily relied on Generalized Lotka-Volterra (gLV) equations. These traditional models successfully laid the theoretical foundation for mapping pairwise species interaction networks and calculating basic carrying capacities. However, their limitations have become increasingly apparent when confronting the highly non-linear, dense metabolic cross-feeding dynamics characteristic of the human microbiome (Dedrick et al., 2023; Mustri et al., 2025).

To overcome this theoretical bottleneck, microbiome dynamic analysis is rapidly integrating cutting-edge Artificial Intelligence algorithms, represented by Neural Ordinary Differential Equations (Neural ODEs) and time-aware Transformer architectures (such as the MicroProphet digital twin framework) (Zhang et al., 2025a,b). These advanced models not only effectively handle the sparsity and irregular sampling challenges typical of longitudinal clinical omics data but can also precisely map and predict individual-specific microbiome evolutionary trajectories with minimal data input. Even more excitingly, by borrowing the theoretical framework of phase transitions in non-equilibrium systems from statistical physics and macroscopic ecology, researchers are striving to apply similar dynamical principles to track “Critical Slowing Down” (CSD) in real-time (Scheffer et al., 2009). Although not originally developed using human microbiome data, this early warning signal—characterized by a sudden drop in recovery rates and surging variance as a system approaches a tipping point—is now being explored to predict dysbiosis.

When these high-dimensional predictive models perfectly fuse with advanced dynamic diagnostic technologies, clinicians will gain unprecedented proactive intervention capabilities. They will be able to accurately predict when a patient is about to fall into a dysbiotic trap, precisely calculate the optimal “seconds” to open a priority-effect intervention window, and tailor highly personalized phage debridement protocols and SMC strain combinations. Ultimately, in this microscopic battle for ecological restoration, rational microbiome engineering will lead us to effectively overcome these pathological barriers and achieve true, enduring host homeostasis.

## References

[B1] Caballero-Flores G. Pickard J. M. Núñez G. (2023). Microbiota-mediated colonization resistance: Mechanisms and regulation. *Nat. Rev. Microbiol.* 21 347–360. 10.1038/s41579-022-00833-7 36539611 PMC10249723

[B2] Cappio Barazzone E. Diard M. Hug I. Larsson L. Slack E. (2024). Diagnosing and engineering gut microbiomes. *EMBO Mol. Med.* 16 2660–2677. 10.1038/s44321-024-00149-4 39468301 PMC11554810

[B3] Chang P. V. (2020). Chemical mechanisms of colonization resistance by the gut microbial metabolome. *ACS Chem. Biol.* 15 1119–1126. 10.1021/acschembio.9b00813 31895538 PMC8218590

[B4] Debray R. Herbert R. A. Jaffe A. L. Crits-Christoph A. Power M. E. Koskella B. (2022). Priority effects in microbiome assembly. *Nat. Rev. Microbiol.* 20 109–121. 10.1038/s41579-021-00604-w 34453137

[B5] Dedrick S. Warrier V. Lemon K. P. Momeni B. (2023). When does a Lotka-Volterra model represent microbial interactions? Insights from in vitro nasal bacterial communities. *mSystems* 8:e0075722. 10.1128/msystems.00757-22 37278524 PMC10308948

[B6] Deter H. S. Lu T. (2022). Engineering microbial consortia with rationally designed cellular interactions. *Curr. Opin. Biotechnol.* 76:102730. 10.1016/j.copbio.2022.102730 35609504 PMC10129393

[B7] Dumas A. Bernard L. Poquet Y. Lugo-Villarino G. Neyrolles O. (2018). The role of the lung microbiota and the gut-lung axis in respiratory infectious diseases. *Cell. Microbiol.* 20:e12966. 10.1111/cmi.12966 30329198

[B8] Fujita H. Yoshida S. Suzuki K. Toju H. (2025). Alternative stable states of microbiome structure and soil ecosystem functions. *Environ. Microbiome* 20:28. 10.1186/s40793-025-00688-4 40050988 PMC11887376

[B9] Garza D. R. Liu B. van de Velde C. Zhou X. Saha P. Gonze D. et al. (2025). Emergence of alternative states in a synthetic human gut microbial community. *Nat. Commun.* 17:326. 10.1038/s41467-025-67036-5 41326405 PMC12789478

[B10] Jones K. R. Song Y. Rinaldi S. S. Moran N. A. (2025). Effects of priority on strain-level composition of the honey bee gut community. *Appl. Environ. Microbiol.* 91:e0082825. 10.1128/aem.00828-25 40742109 PMC12366320

[B11] Khanna S. Sims M. Louie T. J. Fischer M. LaPlante K. Allegretti J. et al. (2022). SER-109: An oral investigational microbiome therapeutic for patients with recurrent *Clostridioides difficile* infection (rCDI). *Antibiotics* 11:1234. 10.3390/antibiotics11091234 36140013 PMC9495252

[B12] Law S. R. Mathes F. Paten A. M. Alexandre P. A. Regmi R. Reid C. et al. (2024). Life at the borderlands: Microbiomes of interfaces critical to one health. *FEMS Microbiol. Rev.* 48:fuae008. 10.1093/femsre/fuae008 38425054 PMC10977922

[B13] Liou M. J. Miller B. M. Litvak Y. Nguyen H. Natwick D. E. Savage H. P. et al. (2022). Host cells subdivide nutrient niches into discrete biogeographical microhabitats for gut microbes. *Cell Host Microbe* 30 836–847.e6. 10.1016/j.chom.2022.04.012 35568027 PMC9187619

[B14] Liu J. Hong W. Sun Z. Zhang S. Xue C. Dong N. (2025). The gut-lung axis: Effects and mechanisms of gut microbiota on pulmonary diseases. *Front. Immunol.* 16:1693964. 10.3389/fimmu.2025.1693964 41562083 PMC12812986

[B15] Liu Q. Tian X. Maruyama D. Arjomandi M. Prakash A. (2021). Lung immune tone via gut-lung axis: Gut-derived LPS and short-chain fatty acids’ immunometabolic regulation of lung IL-1β, FFAR2, and FFAR3 expression. *Am. J. Physiol. Lung Cell. Mol. Physiol.* 321 L65–L78. 10.1152/ajplung.00421.2020 33851870 PMC8321849

[B16] Marrella V. Nicchiotti F. Cassani B. (2024). Microbiota and immunity during respiratory infections: Lung and gut affair. *Int. J. Mol. Sci.* 25:4051. 10.3390/ijms25074051 38612860 PMC11012346

[B17] Maruyama D. Tian X. Doan T. N. M. Liao W. I. Chaki T. Taenaka H. et al. (2026). Gut microbiome-derived propionate reprograms alveolar macrophages metabolically and regulates lung injury responses in mice. *Gut Microbes* 18:2606486. 10.1080/19490976.2025.2606486 41467904 PMC12758369

[B18] McCarty N. S. Ledesma-Amaro R. (2019). Synthetic biology tools to engineer microbial communities for biotechnology. *Trends Biotechnol.* 37 181–197. 10.1016/j.tibtech.2018.11.002 30497870 PMC6340809

[B19] Ming X. Ray C. (2019). Recognizing the effect of ecosystem disruption on human health and neurodevelopment. *Int. J. Environ. Res. Public Health* 16:4908. 10.3390/ijerph16244908 31817321 PMC6950426

[B20] Moreira de Gouveia M. I. Bernalier-Donadille A. Jubelin G. (2024). *Enterobacteriaceae* in the human gut: Dynamics and ecological roles in health and disease. *Biology* 13:142. 10.3390/biology13030142 38534413 PMC10967970

[B21] Mustri M. P. Duan Q. Pawar S. (2025). Accuracy of the Lotka-Volterra model fails in strongly coupled microbial consumer-resource systems. *PLoS Comput. Biol.* 21:e1013719. 10.1371/journal.pcbi.1013719 41337153 PMC12688139

[B22] Ng K. M. Aranda-Díaz A. Tropini C. Frankel M. R. Van Treuren W. O’Loughlin C. T. et al. (2019). Recovery of the gut microbiota after antibiotics depends on host diet, community context, and environmental reservoirs. *Cell Host Microbe* 26 650–665.e4. 10.1016/j.chom.2019.10.011 31726029 PMC8276089

[B23] Pantazi A. C. Balasa A. L. Mihai C. M. Chisnoiu T. Lupu V. V. Kassim M. A. K. et al. (2023). Development of gut microbiota in the first 1000 days after birth and potential interventions. *Nutrients* 15:3647. 10.3390/nu15163647 37630837 PMC10457741

[B24] Pérez Escriva P. Fuhrer T. Sauer U. (2022). Distinct N and C cross-feeding networks in a synthetic mouse gut consortium. *mSystems* 7:e0148421. 10.1128/msystems.01484-21 35357218 PMC9040589

[B25] Rivera-Chávez F. Winter S. E. Lopez C. A. Xavier M. N. Winter M. G. Nuccio S. P. et al. (2013). *Salmonella* uses energy taxis to benefit from intestinal inflammation. *PLoS Pathog.* 9:e1003267. 10.1371/journal.ppat.1003267 23637594 PMC3630101

[B26] Safarchi A. Al-Qadami G. Tran C. D. Conlon M. (2025). Understanding dysbiosis and resilience in the human gut microbiome: Biomarkers, interventions, and challenges. *Front. Microbiol.* 16:1559521. 10.3389/fmicb.2025.1559521 40104586 PMC11913848

[B27] Scheffer M. Bascompte J. Brock W. A. Brovkin V. Carpenter S. R. Dakos V. et al. (2009). Early-warning signals for critical transitions. *Nature* 461 53–59. 10.1038/nature08227 19727193

[B28] Schokker D. Stege P. B. Duhamel M. Bekker M. Timmerman H. M. Kar S. K. et al. (2025). Rationally designed microbial communities in agri-food production systems: From research to market. *ISME Commun.* 5:ycaf121. 10.1093/ismeco/ycaf121 40860564 PMC12376040

[B29] Sencio V. Machado M. G. Trottein F. (2021). The lung-gut axis during viral respiratory infections: The impact of gut dysbiosis on secondary disease outcomes. *Mucosal Immunol.* 14 296–304. 10.1038/s41385-020-00361-8 33500564 PMC7835650

[B30] Singh S. Samson R. Hassard F. (2025). Phage therapy for environmental biotechnology applications. *Front. Microbiol.* 16:1621103. 10.3389/fmicb.2025.1621103 40969428 PMC12442427

[B31] Sleator R. D. Hill C. (2009). Rational design of improved pharmabiotics. *J. Biomed. Biotechnol.* 2009:275287. 10.1155/2009/275287 19753318 PMC2742647

[B32] Spees A. M. Wangdi T. Lopez C. A. Kingsbury D. D. Xavier M. N. Winter S. E. et al. (2013). Streptomycin-induced inflammation enhances *Escherichia coli* gut colonization through nitrate respiration. *mBio* 4:e00430-13. 10.1128/mBio.00430-13 23820397 PMC3705454

[B33] Sprockett D. Fukami T. Relman D. A. (2018). Role of priority effects in the early-life assembly of the gut microbiota. *Nat. Rev. Gastroenterol. Hepatol.* 15 197–205. 10.1038/nrgastro.2017.173 29362469 PMC6813786

[B34] Sun Q. Gao J. Zhao X. Wang T. Pan W. Yu J. (2025). The gut-lung axis in severe pneumonia-related lung injury: Mechanisms and therapeutic strategies. *Front. Immunol.* 16:1700534. 10.3389/fimmu.2025.1700534 41635847 PMC12862065

[B35] Suvvari T. K. Vallurupalli V. Koneru K. S. Ingawale S. Yegurla R. R. (2025). The lasting imprint of antibiotics on gut microbiota: Exploring long-term consequences and therapeutic interventions. *Cureus* 17:e84114. 10.7759/cureus.84114 40519460 PMC12165447

[B36] Van de Guchte M. Burz S. D. Cadiou J. Wu J. Mondot S. Blottière H. M. et al. (2020). Alternative stable states in the intestinal ecosystem: Proof of concept in a rat model and a perspective of therapeutic implications. *Microbiome* 8:153. 10.1186/s40168-020-00933-7 33158453 PMC7646066

[B37] Verma A. Bhagchandani T. Rai A. Nikita, Sardarni U. K. Bhavesh N. S. et al. (2024). Short-Chain Fatty Acid (SCFA) as a connecting link between microbiota and gut-lung axis-a potential therapeutic intervention to improve lung health. *ACS Omega* 9 14648–14671. 10.1021/acsomega.3c05846 38585101 PMC10993281

[B38] Wang Z. Yu J. Liu Y. Gong J. Hu Z. Liu Z. (2025). Role of the microbiota-gut-lung axis in the pathogenesis of pulmonary disease in children and novel therapeutic strategies. *Front. Immunol.* 16:1636876. 10.3389/fimmu.2025.1636876 41080577 PMC12507888

[B39] Winter S. E. Winter M. G. Xavier M. N. Thiennimitr P. Poon V. Keestra A. M. et al. (2013). Host-derived nitrate boosts growth of *E. coli* in the inflamed gut. *Science* 339 708–711. 10.1126/science.1232467 23393266 PMC4004111

[B40] Xue K. S. Walton S. J. Goldman D. A. Morrison M. L. Verster A. J. Parrott A. B. et al. (2023). Prolonged delays in human microbiota transmission after a controlled antibiotic perturbation. *bioRxiv* [Preprint] 10.1101/2023.09.26.559480 37808827 PMC10557656

[B41] Zhang Y. Zhou K. Chen X. Zhang H. Han J. Ning K. (2025a). A temporal-aware machine learning framework enables microbial community dynamics prediction with personalized precision. *Microbiome* 13:261. 10.1186/s40168-025-02269-6 41462375 PMC12751615

[B42] Zhang Y. Zhou K. Chen X. Zhang H. Han J. Ning K. (2025b). MicroProphet: A digital twin framework for predicting microbial community dynamics with personalized precision. *bioRxiv* [Preprint] 10.1101/2025.05.08.652793 PMC12751615 41462375

